# Mechanism-Based Pharmacokinetic Model for the Deglycosylation Kinetics of 20(S)-Ginsenosides Rh2

**DOI:** 10.3389/fphar.2022.804377

**Published:** 2022-05-25

**Authors:** Hong-can Ren, Jian-guo Sun, Ji-ye A, Sheng-hua Gu, Jian Shi, Feng Shao, Hua Ai, Jing-wei Zhang, Ying Peng, Bei Yan, Qing Huang, Lin-sheng Liu, Yang Sai, Guang-ji Wang, Cheng-guang Yang

**Affiliations:** ^1^ Key Lab of Drug Metabolism and Pharmacokinetics, State Key Laboratory of Natural Medicines, China Pharmaceutical University, Nanjing, China; ^2^ DMPK and Clinical Pharmacology Group, Hutchison MediPharma Ltd., Shanghai, China; ^3^ Department of Biology, GenFleet Therapeutics, Shanghai, China; ^4^ School of Pharmacy, Shanghai University of Tranditional Chinese Medicine, Shanghai, China; ^5^ College of Pharmacy, University of Michigan, Ann Arbor, MI, United States; ^6^ NMPA Key Laboratory for Impurity Profile of Chemical Drugs, Jiangsu Institute for Food and Drug Control, Nanjing, China; ^7^ Department of Pharmacy, The First Affiliated Hospital of Soochow University, Suzhou, China; ^8^ Department of General Surgery, Tongren Hospital, Shanghai Jiao Tong University School of Medicine, Shanghai, China

**Keywords:** traditional Chinese medicine, ginsenosides, pharmacokinetics, deglycosylation, modelling and simulation

## Abstract

**Aim:** The 20(S)-ginsenoside Rh2 (Rh2) is being developed as a new antitumor drug. However, to date, little is known about the kinetics of its deglycosylation metabolite (protopanoxadiol) (PPD) following Rh2 administration. The aim of this work was to 1) simultaneously characterise the pharmacokinetics of Rh2 and PPD following intravenous and oral Rh2 administration, 2) develop and validate a mechanism-based pharmacokinetic model to describe the deglycosylation kinetics and 3) predict the percentage of Rh2 entering the systemic circulation in PPD form.

**Methods:** Plasma samples were collected from rats after the I.V. or P.O. administration of Rh2. The plasma Rh2 and PPD concentrations were determined using HPLC-MS. The transformation from Rh2 to PPD, its absorption, and elimination were integrated into the mechanism based pharmacokinetic model to describe the pharmacokinetics of Rh2 and PPD simultaneously at 10 mg/kg. The concentration data collected following a 20 mg/kg dose of Rh2 was used for model validation.

**Results:** Following Rh2 administration, PPD exhibited high exposure and atypical double peaks. The model described the abnormal kinetics well and was further validated using external data. A total of 11% of the administered Rh2 was predicted to be transformed into PPD and enter the systemic circulation after I.V. administration, and a total of 20% of Rh2 was predicted to be absorbed into the systemic circulation in PPD form after P.O. administration of Rh2.

**Conclusion:** The developed model provides a useful tool to quantitatively study the deglycosylation kinetics of Rh2 and thus, provides a valuable resource for future pharmacokinetic studies of glycosides with similar deglycosylation metabolism.

## 1 Introduction

Ginseng is a traditional medicine that has been used for centuries. The global ginseng extracts market size was valued at USD 22.9 billion in 2019 and is expected to grow at a compound annual growth rate (CAGR) of 6.2% from 2020 to 2027 (Future Market Insights, 2020). Ginsenosides, a class of natural product steroid glycosides, have a wide effect on the cardiovascular system, central nervous system and immune system (Wang et al., 2014; Zhang et al., 2019a; Alolga et al., 2020; Zhu et al., 2020). Recent studies have found that 20(S)-ginsenosides Rh2 (Rh2), a substance isolated from red ginseng, may inhibit the growth of various cancer cells, reverse sleep deprivation-induced cognitive deficit, improve insulin sensitivity, and, enhance the antitumor immunological response in a melanoma mice model (Lee et al., 2007; Wang et al., 2017; Lu et al., 2018; Zhang et al., 2019b; Jeong et al., 2019; Qi et al., 2019).

**FIGURE 1:**
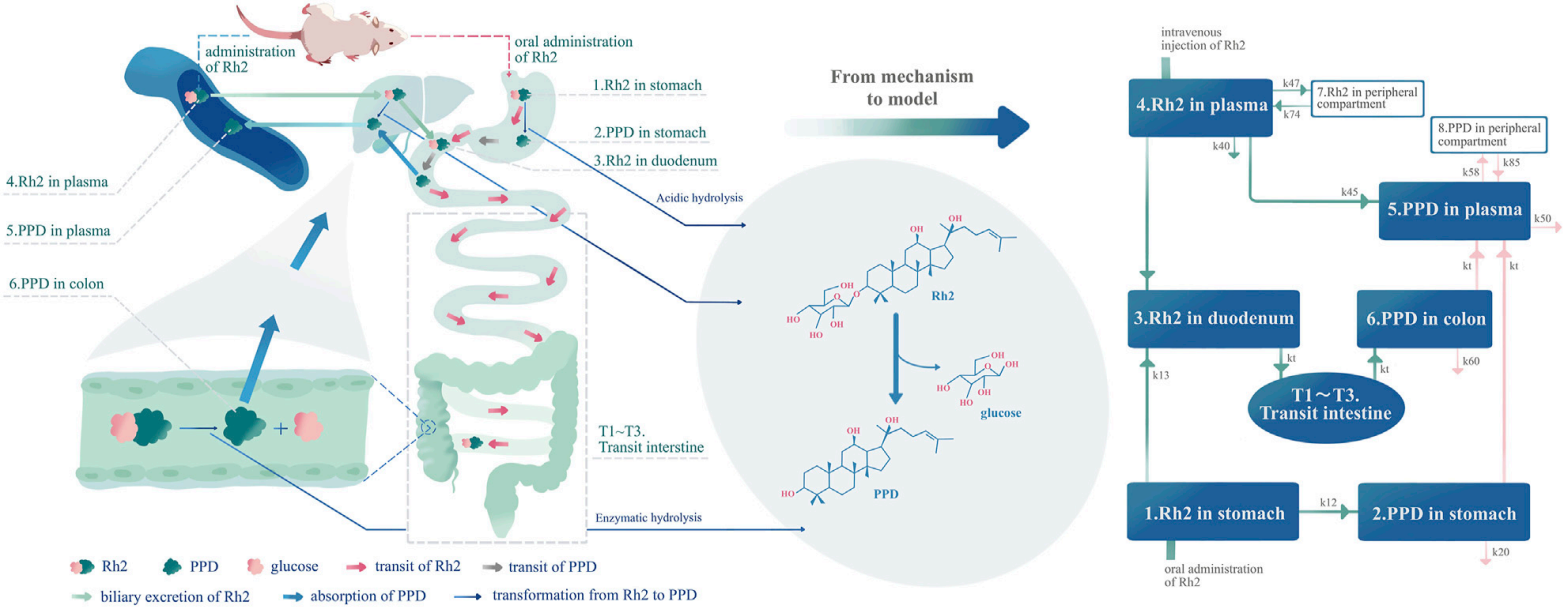
Development of the pharmacokinetic model according to the mechanism of the deglycosylation kinetics of Rh2 and *in vivo* metabolite (PPD) kinetics in rats. The left part of this figure is the mechanism diagram corresponding to the structure of pharmacokinetic model (right part of the figure). Hydrolysis in the liver, transformation from Rh2 to PPD in the liver (k_45_), route A in text article. Route A is assumed to exist since the PPD can be detected immediately after I.V. dosing of Rh2. Enzymatic hydrolysis, transformation from Rh2 to PPD by glycoside hydrolases from microflorae in the colon (k_t_ from compartment T3 to compartment 6) (Qian and Cai, 2010), route B in the text article. Acidic hydrolysis, transformation from Rh2 to PPD by stomach acid (k_12_) (Bae et al., 2004), route C in text article.

However, the permeability of Rh2 in Caco-2 cells has been reported to be low, and it has also been reported to exhibit poor absolute bioavailability in rats (Qian et al., 2005; Gu et al., 2010). Here, Rh2’s poor bioavailability and permeability do not appear to support its *in vivo* bioactivity. One of the hypotheses is that Rh2 is bio-transformed by gut microbiota, thereby producing new bioactive molecules with better absorption to exert the bioactivity (Gong et al., 2020). Rh2 is a protopanaxadiol (PPD)-type ginsenoside; this type has one glucose moiety at the C3 hydroxyl of PPD as shown in Figure 1. As the second genome of the body, the microbiome involves the metabolism of many drugs (Zimmermann et al., 2019; Brody, 2020; Savage, 2020). The glycosidase activities present in the human colonic microbiota act on many glycosides including Rh2 (Dabek et al., 2008; Gloster et al., 2008). For example, the bioavailability of ginsenoside Rb1 has been reported to be low (Akao et al., 1998). Only the primary deglycosylation metabolite of ginsenoside Rb1 (compound K) could be detected in plasma, where its concentrations were found to be retained in the plasma for a long period of time following administration of ginsenoside Rb1 (Akao et al., 1998).

This hypothesis is further supported by the following evidence reported in the literature: 1) Rh2 can be deglycosylated into its metabolite, PPD, in the gastrointestinal tract (Bae et al., 2004), 2) the bioavailability of PPD is 36.8%, a bioavailability much greater than that seen in rats (Ren et al., 2008), and, 3) PPD is an active metabolite that demonstrates a potency in inducing apoptosis, altering membrane integrity, and inhibiting triple-negative breast cancer metastasis (Popovich and Kitts, 2002; Peng et al., 2019). However, to date, there is no direct evidence from any *in vivo* pharmacokinetic study to further validate this hypothesis and provide a comprehensive understanding of the kinetic profiles of Rh2 and its active metabolite.

The above studies provided the knowledge with regard to the pharmacokinetics of Rh2 and its active metabolite PPD, which could be integrated into a mechanism based pharmacokinetic model to study the deglycosylation kinetics of Rh2. As shown in Figure 1, the mechanism based pharmacokinetic model included all the possible deglycosylation kinetics of Rh2 in plasma (k_45_), stomach (k_12_), and colon (k_t_). The classical two-compartment pharmacokinetic model was included in the mechanism based pharmacokinetic model to describe the distribution and elimination of Rh2 (compartment 4 and 7) and PPD (compartment 5 and 8). In doing so, it could facilitate the pharmacokinetic/pharmacodynamic (PK/PD) analysis of ginseng. On the other hand, Rh2 has been reported to be a potent non-competitive P-gp inhibitor (Gu et al., 2010). This has led to the concern that a potential herb-drug interaction may exist between ginseng and drugs that are P-gp substrates (Zhang et al., 2010). The mechanistic pharmacokinetic model of Rh2 and PPD would be the starting point to quantitatively assess the potential existence of the herb-drug interaction.

The aim of this study was to gain a better understanding of the deglycosylation kinetics of 20(S)-Ginsenosides Rh2 by integrating experimental data with prior biological mechanism knowledge. To achieve the aim, the study comprised of the following three specific objectives: 1) to simultaneously characterise the pharmacokinetics of Rh2 and PPD following intravenous (I.V.) and oral (P.O.) Rh2 administration, 2) to develop a mechanism-based pharmacokinetic model based on the hypothesised deglycosylation kinetics and 3) predict the percentage of Rh2 elimination by gut bacteria using the developed model.

## 2 Materials and Methods

The methods section is described in two parts. The first part provides the technical details of the animal experiment and sample analysis. The second part describes the details of the data analysis, which includes: 1) an exploratory data analysis, 2) the development of a mechanism-based model for the deglycosylation of glycosides *in vivo*, and 3) a quantitative assessment of the contribution of each elimination route.

### 2.1 Part I

#### 2.1.1 Chemical and Reagents

20(S)-Ginsenosides Rh2 (Rh2), PPD and panoxadiol (purity over 99%) were purchased from the Department of Nature Medical Chemistry, School of Chemistry, Jilin University, Changchun, China. Deionized water was prepared using the Milli-Q system (Millipore, Bedford, MA, United States). Methanol of HPLC grade was purchased from Merck, Darmstadt Germany. Acetic ether and all other reagents (including solvents) were of analytical grade. Pure nitrogen gas was supplied by the Gas Supplier Center of Nanjing University, China.

#### 2.1.2 Animal Experiments

Young adult Sprague–Dawley male rats with a body weight of 240–280 g were purchased from Sino-British Sippr/BK Lab Animal Ltd. (Shanghai, China). This research study and all animal handling procedures were approved by Research Animal Care and Laboratory Animal Resources of China Pharmaceutical University. The animal studies adhered to the European Community guidelines for laboratory animal care.

Prior to commencing the experiment, the rats were given 1 week to acclimatise to the animal facility. Rh2 was dissolved in saline with 50% hydroxypropyl-β-cyclodextrin for I.V. or P.O. administration–a volume of 5 ml/kg was given. Twelve rats were divided into four groups. Following an overnight fast (of at least 12 h), rats allocated to groups 1 and 2 (*n* = 3 per group) were administered an I.V. bolus of 10 mg/kg and 20 mg/kg of Rh2, respectively; whilst rats allocated to groups 3 and 4 received a 10 and 20 mg/kg oral dose of Rh2, respectively (*n* = 3 per group). The 20(S)-Ginsenosides Rh2 occurred as O-glycosides with glucose bound in nature, which could be hydrolysed by β-O-glycosidase (Zheng et al., 2017). The activity of the glycosidase was not affected by sex difference significantly in mice as reported in a previous study (Doonan et al., 1978). Therefore, t authors assumed that the sex difference might have little effect on studying the transformation of PPD in this study. Herein, only males were included in this study. The relatively high dose levels were given to rats considering the poor bioavailability of Rh2. As the data were analysed by simultaneous global fitting of concentrations from the parent drug and metabolite at multiple time points, the effective sample size is far larger than experimental replication. In addition, no statistical comparison was performed on these data; rather, they were used for the development of a kinetic model of deglycosylation. Blood samples were collected by orbital sinus bleeding at 0 (prior to dosing), 0.25, 0.50, 1, 2, 4, 8, 12, 16, 20 (or 24) h post-drug administration. Water was freely accessible throughout the study, however access to food was restricted until 3 h post-drug administration. The collected samples were centrifuged at 4,000 rpm for 5 min. The separated plasma samples were then stored at -80°C.

#### 2.1.3 Sample Preparation

Ten microliters of panoxadiol (2.0 μg/ml) were added to 0.1 ml of plasma creating an internal standard. This procedure was followed by a liquid–liquid extraction using 1.0 ml of acetic ether. The organic and aqueous phases of plasma were separated by centrifugation at 8,000 rpm for 5 min. The upper organic phase was transferred to another tube and evaporated using a Thermo Savant SPD 2010 Speed Vac System (Thermo Electron Corporation, United States) set at 40°C. The residue was reconstituted into 100 µl of the mobile phase using a vortex for 1 min. After centrifugation at 20,000 rpm for 10 min, 5 µl of the solution was injected into the column.

#### 2.1.4 HPLC-MS Analysis for Determination of Plasma Concentrations

The method used to determine plasma concentration PPD has previously been published by Ren et al. (Ren et al., 2008). The HPLC method was revised for elution of the simultaneous determination of plasma Rh2 and PPD concentrations. Details of the method and validation are summarised in the Supporting Information.

### 2.2 Part II

#### 2.2.1 Exploratory Data Analysis

A non-compartmental analysis (NCA) was performed to determine the following pharmacokinetic metrics for Rh2 and PPD: C_max_, T_max_, V_ss_, and T_1/2_. The NCA was performed using Phoenix 64 Winnonlin (Pharsight, a Certara™ Company, Cary, NC, United States). For Rh2 the I.V. dosing option was selected, whilst the extravascular dosing option was selected for PPD.

#### 2.2.2 Workflow of Model Development and Validation

A brief workflow describing model development and validation is presented as follows:

(i) The known mechanism of deglycosylation kinetics (Figure 1) was summarised to explain the formation and *in vivo* kinetics of the metabolite (PPD). A mechanism based pharmacokinetic model was then implemented (as shown in Figure 1).
(ii) Based on the method of drug administration the proposed mechanistic model was further reduced into four sub-models (i.e., A, B, C, and D) for the estimation of model parameters. The observed data following I.V. or P.O. administration of Rh2 at 10 mg/kg was used for model fitting. Details of the sub-models are introduced in Appendix A. The model fitting results of the sub-models are provided in Supporting Information.
(iii) The concentration versus time data of Rh2 and PPD at 20 mg/kg was used for external validation of the developed mechanistic model.

#### 2.2.3 Overview of Mechanistic-Based Pharmacokinetic Model

As shown in Figure 1, the model accounts for the distribution (i.e., transit in the intestine) and elimination of Rh2, as well as the formation, absorption, distribution, and elimination of PPD. The proposed model can describe the concentration time profiles of both Rh2 and PPD simultaneously after I.V. and/or P.O. administration of Rh2 in rats. Following the I.V. administration of Rh2 it is eliminated by three routes: 1) direct transformation from Rh2 to PPD (k_45_), 2) biliary excretion (k_43_), and 3) other unknown routes (k_40_) in the systemic circulation. Following the P.O. administration of Rh2 it is eliminated by two routes: 1) transformation of Rh2 to PPD in the stomach (k_12_), 2) transfer from stomach to colon through the transit compartments T1-T3 and metabolism in colon. The transformation of Rh2 to PPD in the colon is known to be the primary route of formation for PPD. The absorption of PPD in the stomach (it may be transferred to the duodenum and then absorbed) and colon into the systemic circulation is described by k_t_, whilst its elimination is described by the rate constant k_50_. The peripheral compartments (compartment 7 and 8) are implemented to describe the distribution of Rh2 and PPD into the peripheral tissues, respectively. A detailed description of the model is provided in Appendix A.1. The model can be further reduced into sub-models to describe the concentration time profiles of Rh2 and PPD following I.V. and P.O. administration of Rh2 respectively (see in Section 2.2.5).

#### 2.2.4 Software and Criteria for Model Development

The mechanism-based pharmacokinetic model was developed using the non-linear mixed effect modelling software Phoenix 64 NLME (version 8.2, Pharsight, a Certara™ Company, Cary, NC). Inter-individual variability was described using an exponential model. Residual error was described by a multiplicative error model. The initial estimates used in the model were obtained by manual adjustment of parameters and visual inspection. The FOCE ELS algorithm in Phoenix 64 NLME was used for parameter estimation. The code for the final model is provided in Supporting Information. The model was evaluated based on successful convergence, objective function value, parameter precision, visual inspection of goodness-of-fit plots and a visual predictive check (VPC) (Wang and Zhang, 2012).

#### 2.2.5 Strategy of the Parameter Estimation

The final model was comprised of 11 compartments and 16 parameters. This led to challenges in model parameter estimation. Hence, to reduce computational workload, a model reduction strategy was applied using the following four steps:

Step 1: The full model was reduced to sub-model A, including compartments of “4. Rh2 in plasma” and “7. Rh2 in PC”. The parameters related to the elimination and distribution of Rh2 were then estimated using the raw data following I.V. administration of Rh2. Details for the sub-model A are provided in Appendix A.

Step 2: The full model was reduced to sub-model B including compartments of “5. Rh2 in plasma” and “8. Rh2 in PC”. Similar to sub-model A for the independent modelling of Rh2, the parameters related to the elimination and distribution of PPD were estimated using the raw data following I.V. administration of PPD for the independent modelling of PPD.

Step 3: Sub-model C was comprised of the following components: sub-model A, sub-model B, biliary excretion, transit of Rh2, transformation of Rh2 to PPD in the colon and absorption of PPD. The parameters were then estimated using the reported biliary excretion data of Rh2 and the concentration-time profile of PPD following a 10 mg/kg I.V. administered dose of Rh2 (Gu et al., 2009).

Step 4: Sub-model D was comprised of the following components: sub-model B, transit of Rh2 from the stomach to the intestine, transformation of Rh2 to PPD in the stomach, and elimination of PPD in the intestine compartments (linked by blue and purple arrows in Figure 1). The related parameters were estimated using plasma concentrations of PPD following a 10 mg/kg dose of Rh2 administered P.O.

Details for each of the sub-models is provided in Appendix A.

#### 2.2.6 External Validation

The model was externally validated by performing a visual predictive check (VPC) (Wang and Zhang, 2012). The model parameters were fixed to simulate the concentration-time profiles for Rh2 and PPD following I.V. and P.O. administration when given at a 20 mg/kg dose. The 5^th^, 50th, and 95th percentiles were calculated from the empirical posterior distribution of 1000 replicates. If the majority of observed concentration data was within the 90% prediction interval the model was thought to be validated.

#### 2.2.7 Evaluation of Elimination Contribution by Different Routes

The final model was used to quantitatively assess the contribution of each elimination route of Rh2. Following I.V. administration, Rh2 is eliminated via three different routes which are: 1) transformation from Rh2 to PPD in the plasma or liver (route A), 2) biliary excretion, transformation from Rh2 to PPD, and re-absorption into the systemic circulation (route B), and 3) other unknown metabolism routes. We assumed that route A exists without robust evidence since the PPD could be detected immediately after IV dosing of Rh2. The extent of Rh2 transformation to PPD in plasma (route A) was quantified as the ratio of direct transformation rate constant from Rh2 to PPD to the total elimination rate constant of Rh2 (k_45_/k_e_). The excreted Rh2 by bile was partly transformed into PPD in the colon and further re-absorbed into the systemic circulation (route B). The extent of biliary excretion was quantified as the ratio of the biliary excretion rate constant to the total elimination rate constant of Rh2 (k_43_/k_e_). The extent of re-absorbed PPD was the product of the percentage of biliary excretion and the percentage of PPD absorbed from the colon to the systemic circulation (k_43_/k_e_ ∙ k_t_/(k_t_ + k_60_). The extent of the other unknown elimination routes was quantified as the sum of unknown elimination of Rh2 in plasma and bile (k_40_/k_e_ + k_43_/k_e_ ∙ k_60_/(k_t_ + k_60_)) or 100% minus the percentage of Rh2 entering the systemic circulation in PPD form (100% - k_45_/k_e_ - k_43_/k_3_ ∙ k_t_/(k_t_ + k_60_)). After P.O. administration, Rh2 is transformed to PPD in the stomach and colon via metabolism. The total disposition rate constant of Rh2 in the stomach is the sum of the transit rate constant from the stomach to the intestine and the transformation rate constant from Rh2 to PPD in the stomach (k_12_ + k_13_). The extent of Rh2 transiting from the stomach to the intestine was quantified as the ratio of transit rate constant from the stomach to the intestine and the total disposition of Rh2 in the stomach, expressed as k_13_/(k_12_ + k_13_). The extent of Rh2 metabolism in the stomach was expressed as k_12_/(k_12_ + k_13_). PPD present in the stomach can be transferred to the duodenum and absorbed into the systemic circulation, which has been simplified as absorption rate constant (k_t_), or eliminated via unknown mechanisms (k_20_). Hence, the total disposition rate constant of PPD in the stomach is the sum of both of the routes (k_t_ + k_20_). The extent of PPD absorption in the stomach was quantified as the ratio of absorption rate constant of PPD to total disposition rate constant of PPD: k_t_/(k_t_ + k_20_). The extent of PPD elimination in the stomach was quantified as the ratio of elimination rate constant of PPD to total disposition rate constant of PPD in the stomach: k_20_/(k_t_ + k_20_). The percentage of Rh2 transformed to PPD in the colon and further absorbed into the systemic circulation (route B) was the product of the percentage of Rh2 transiting from the stomach to the intestine and the percentage of PPD absorbed from the colon to the systemic circulation (k_13_/(k_12_ + k_13_) ∙ k_t_/(k_t_ + k_60_)). The percentage of Rh2 transformed to PPD in the stomach and absorbed into the systemic circulation (route C), was the product of the percentage of Rh2 transiting from the stomach to the intestine and the percentage of PPD absorption in the stomach: k_12_/(k_12_ + k_13_) ∙ k_t_/(k_t_ + k_20_). The extent of the other unknown elimination routes was quantified as the sum of unknown elimination of Rh2 in the stomach and colon (k_13_/(k_12_ + k_13_) ? k_60_/(k_t_ + k_60_) + k_12_/(k_12_ + k_13_) ? k_20_/(k_t_ + k_20_)) or 100% minus the percentage of Rh2 entering the systemic circulation in PPD form (100% - k_13_/(k_12_ + k_13_) ∙ k_t_/(k_t_ + k_60_) - k_12_/(k_12_ + k_13_) ∙ k_t_/(k_t_ + k_20_)).

## 3 Results

### 3.1 The Result of Exploratory Data Analysis

A summary of the non-compartmental analysis results is presented in Table 1. The pharmacokinetic profile of PPD following an I.V. or P.O. dose of Rh2 were characterised by an atypical double peak. Following an I.V. or P.O. dose of Rh2, PPD reached a maximum concentration by approximately 6 h post Rh2 administration. The T_max_ of PPD was longer than that after only oral dosing of PPD (2.5 h), indicating a long delay of transformation from Rh2 to PPD after dosing of Rh2. The PPD has high exposure after administration of Rh2: the exposure of PPD (AUC_0-t_, 1039 h nmol/L) is around 71% of the exposure of Rh2 (AUC_0-t_, 1457 h nmol/L) after I.V. administration of Rh2 at a dose level of 10 mg/kg. At an oral Rh2 dose of 10 mg/kg the concentration of Rh2 was lower than the lower limit of quantitation (LLOQ), though the AUC for PPD was 2,377 h nmol/L. This was obviously higher than that after I.V. of Rh2 at the same dose in rats. The C_max_ and AUC_0-t_ of Rh2 and PPD were found to increase proportionally with the dose. The other PK parameters are shown in the Supporting Information.

**TABLE 1:** The pharmacokinetic parameters from non-compartment analysis.

Determination of	Parameters	10 mg/kg		20 mg/kg	
		Mean	CV (%)	Mean	CV (%)
I.V. administration of Rh2					
Rh2	V_ss_ (L/kg)	17.1	86.9	20.2	77.1
	AUC_0-t_ (h∙nmol/L)	1457	42.2	3,850	77.5
	t_1/2_ (h)	2.23	10.6	2.36	29.0
PPD	C_max_ (nmol/L)	94.9	22.9	174	31.7
	T_max_ (h)	6.83	85.4	6.75	88.5
	AUC_0-t_ (h∙nmol/L)	1039	37.7	2,827	40.5
P.O. administration of Rh2					
PPD	C_max_ (nmol/L)	255	17.1	442	49.5
	T_max_ (h)	8.00	0.00	10.7	21.7
	AUC_0-t_ (h∙nmol/L)	2,377	15.3	4,611	44.8

### 3.2 Kinetic Modelling of Rh2 and PPD After I.V. Administration (Sub-model A and B)

**FIGURE 2:**
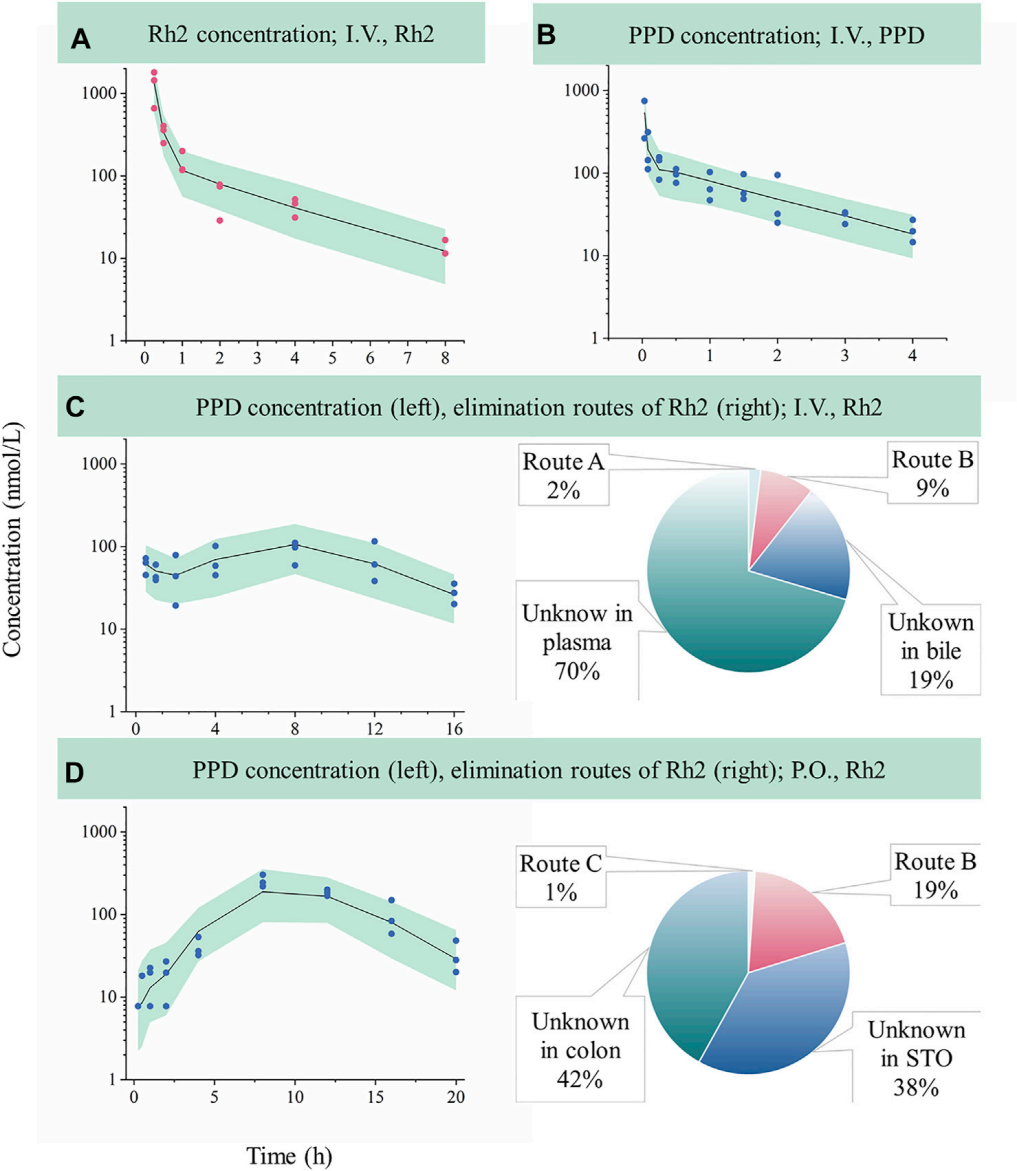
Evaluation of model performance in rats and the calculated elimination routes of Rh2. Green areas represent the 90% confidence interval between the 5th and 95th of percentiles, solid lines are the median profile (50th point of percentile), red symbols represent raw observations of Rh2, and blue symbols represent raw observations of PPD. **(A)**, Rh2 pharmacokinetic profile after I.V. administration of Rh2 at 10 mg/kg; **(B)**, PPD pharmacokinetic profile after I.V. administration of PPD at 0.2 mg/kg; **(C)**, PPD pharmacokinetic profile after I.V. administration of Rh2 at 10 mg/kg; **(D)**, PPD pharmacokinetic profile after P.O. administration of Rh2 at 10 mg/kg. Route A is the percentage of the administered Rh2 transformed from Rh2 to PPD in the systemic circulation; Route B is the percentage of the administered Rh2 transformed to PPD in the colon and absorbed into the systemic circulation; Route C is the percentage of the administered Rh2 transformed to PPD in the stomach and absorbed into the systemic circulation. STO refers to stomach.

Following an I.V. dose, the pharmacokinetic profiles of Rh2 and PPD were characterized by a rapid drop in plasma concentration followed by a relative slower decrease in the terminal phase. This conforms with the features of a classic two-compartment model. Sub-models A and B provided a reasonable description of the observed data (shown in Figures 2A,B). The parameters of sub-models A and B were precisely estimated (as shown in Table 2). The elimination rate constant of Rh2 (k_e_) was estimated to be 4.67 h^−1^, a value similar to the elimination rate constant of PPD (k_50_,4.88 h^−1^). This indicates a similar *in vivo* elimination in rats. The distribution of Rh2 was notably different to PPD. This was evident by the large differences between the distribution related parameters of Rh2 (k_47_, k_74_, and V_Rh2, plasma_) and the corresponding parameters for PPD (k_58_, k_85_, and V_PPD, plasma_). These model parameters were fixed when estimating the parameters in sub-models C and D.

**TABLE 2:** Summary of estimated pharmacokinetic parameters.

Parameters	Definition	Estimate	CV%	95% CI	
				Lower	Upper
Rh2 independent parameters after I.V. administration of Rh2					
V_Rh2, plasma_ (L/kg)	Volume of central compartment distribution of Rh2	2.39	16.4	1.54	3.23
K_e_ (1/h)	Elimination rate constant of Rh2 in central compartment	4.67	3.90	4.28	5.07
K_47_ (1/h)	Transfer rate constant of Rh2 from central compartment to peripheral compartment	2.08	14.8	1.42	2.75
K_74_ (1/h)	Transfer rate constant of Rh2 from peripheral compartment to central compartment	0.48	15.0	0.32	0.63
PPD independent parameters after I.V. administration of PPD					
V_PPD, plasma_ (L/kg)	Volume of central compartment distribution of PPD	0.29	27.0	0.13	0.44
K_50_ (1/h)	Elimination rate constant of PPD in central compartment	4.88	29.3	1.99	7.77
K_58_ (1/h)	Transfer rate constant of PPD from central compartment to peripheral compartment	27.3	19.5	16.5	38.0
K_85_ (1/h)	Transfer rate constant of PPD from peripheral compartment to central compartment	3.38	9.68	2.72	4.04
PPD parameters after I.V. administration of Rh2					
K_45_ (1/h)	Transformation rate constant from Rh2 to PPD in systemic circulation	0.09	12.9	0.07	0.12
K_43_ (1/h)	Excretion rate constant of Rh2 in bile	1.29	NA	NA	NA
K_40_ (1/h)	Elimination rate constant of Rh2 by other routes in systemic circulation	3.29	NA	NA	NA
K_60_ (1/h)	Elimination rate constant of PPD in colon	1.38	16.1	0.90	1.85
K_t_ (1/h)	Transit rate constant of Rh2 in intestines	0.63	7.53	0.53	0.73
PPD parameters after P.O. administration of Rh2					
K_13_ (1/h)	Transit rate constant of Rh2 from stomach to duodenum	0.22	9.66	0.17	0.26
K_12_ (1/h)	Transformation rate constant from Rh2 to PPD in stomach	0.14	14.5	0.10	0.18
K_20_ (1/h)	Elimination rate constant of PPD in stomach	22.2	20.6	12.6	31.8

NA, not available. The estimates of k_43_ and k_40_ have been frozen. CI, confidence interval.

### 3.3 Kinetic Modelling of PPD After I.V. Administration of Rh2 (Sub-Model C)

Sub-model C provided a good description of the concentration-time course of PPD following an I.V. administration of Rh2 (shown in Figure 2C). This was shown by the majority of observations lying within the 90% confidence interval in Figure 2C. This finding was consistent with the visual inspection of the goodness-of-fit plots (shown in Figure S1 in Supporting Information). As shown in Table 2, the parameters in sub-model C were precisely estimated with CV values below 30%. The value for k_e_ (4.67 h^−1^) was determined by summing k_43_ (1.29 h^−1^), k_40_ (3.29 h^−1^), and k_45_ (0.091 h^−1^). Here, it was found that 2% of the administered Rh2 was eliminated via direct transformation from Rh2 to PPD in plasma. Around 28% of the administered Rh2 was eliminated via biliary excretion among which 9% of the administered Rh2 were transferred into the colon, transformed into PPD, and then absorbed into the systemic circulation. The other 19% of the administered Rh2 in bile and 70% of the administered Rh2 in plasma were eliminated via unknown routes.

### 3.4 Kinetic Modelling of PPD After P.O. Administration of Rh2 (Sub-Model D)

Sub-model D provided a good description of the time course of PPD following P.O. administration of Rh2. This was shown by most of the observations lying within the 90% prediction interval shown in Figure 2D. This was consistent with the visual inspection of the goodness-of-fit plots (presented in Supporting Information). As shown in Table 2, the parameters in sub-model D were precisely estimated with RSE values below 30%. Around 61% of the administered Rh2 transited from the stomach into the colon among which 19% of the administered Rh2 was followed by deglycosylation, which might explain the high exposure of PPD following P.O. as opposed to I.V. administration when the same dose was given. Only 1% of the administered Rh2 was metabolised to PPD in the stomach and absorbed into the circulatory system as PPD. In total, 80% of administered Rh2 was eliminated by other unknown routes.

### 3.5 Model External Validation

**FIGURE 3:**
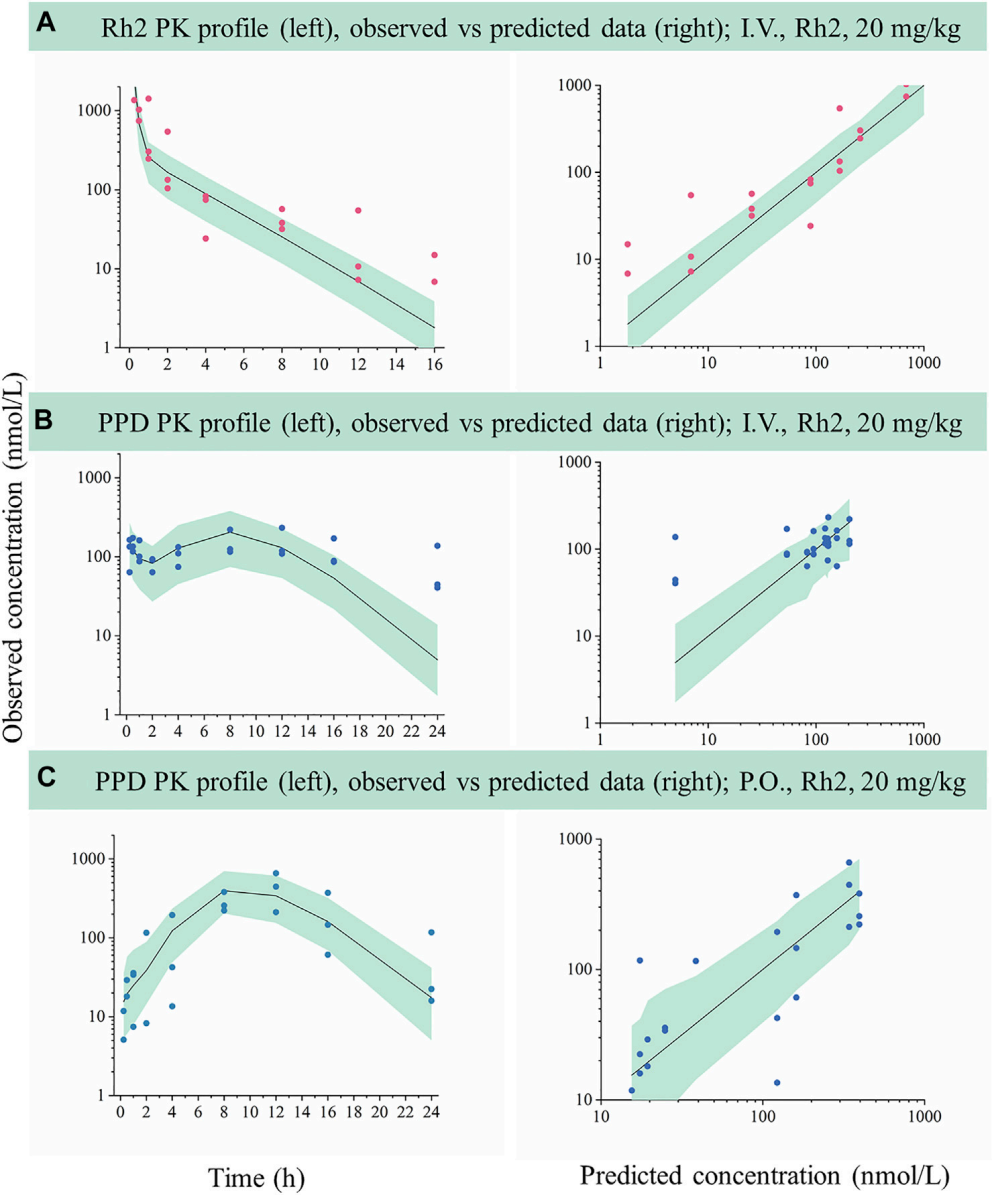
External evaluation of the developed mechanistic pharmacokinetic model using the observed data from 20 mg/kg groups. Green areas represent the 90% model prediction interval between the 5th and 95th of percentiles, solid lines are the median model prediction (50th point of percentile), red symbols represent raw observations of Rh2, and blue symbols represent raw observations of PPD. **(A)**, Rh2 pharmacokinetic profile after I.V. administration of Rh2 at 20 mg/kg; **(B)**, PPD pharmacokinetic profile after I.V. administration of Rh2 at 20 mg/kg; **(C)**, PPD pharmacokinetic profile after P.O. administration of Rh2 at 20 mg/kg. The 90% model prediction intervals were predicted by the model developed by the data at 10 mg/kg. Most of the observed Rh2 or PPD concentrations lay within the predicted 5th and 95th percentiles with the majority of observations being evenly distributed around the median.

The developed model based on data following a 10 mg/kg dose was used to predict the concentration versus time profiles for a 20 mg/kg dose. The predicted data were compared with the observed data for external validation via a VPC. As shown in Figure 3, most of the observed Rh2 or PPD concentrations was within the predicted 5th and 95th percentiles with the majority of observations being evenly distributed around the median. Hence, the results indicated that the developed model could describe the observed data when predicting a higher dose.

## 4 Discussion

In recent years, the interaction between the bioactive ingredients of traditional Chinese medicine (TCM) and gut microbiota has attracted much attention (Laparra and Sanz, 2010; Dey, 2019; Feng et al., 2019; Yue et al., 2019; Gong et al., 2020; Jia et al., 2020). The bio-transformation by gut microbiota and their effect on the pharmacokinetics and therapeutic role have been reported (Choi et al., 2018; Bridgeman et al., 2020; Savage, 2020). However, few reports used modelling and simulation to quantitatively study the bio-transformation from parent drug to metabolite. In this study, the parent drug and deglycosylation metabolite were detected simultaneously after dosing of Rh2 in rats and a model-based method was used for the study of bio-transformation with minimum data requirements. As a result, high concentrations of PPD were detected following the administration of Rh2. This highlights the importance of simultaneously investigating the pharmacokinetic profiles of Rh2 and PPD. Following I.V. or P.O. administration of Rh2, the pharmacokinetic profile of PPD showed atypical double peaks with a prolonged T_max_. The mechanisms of PPD formation were summarized and the hypothesis of deglycosylation kinetics was proposed in Figure 1 to qualitatively explain the formation and *in vivo* kinetics of the metabolite (PPD) based on the reported publications. The mechanism of deglycosylation kinetics involved the transformation from parent drug (Rh2) to metabolite (PPD) by acid in the stomach and by microflorae in the colon, as well as the transit of Rh2 and PPD in intestinal tracts, and biliary excretion of Rh2. Since the liver concentrations were not measured, we did not include a separate liver compartment in the final model; instead it was merged into the plasma compartment.

The mechanism-based pharmacokinetic model could provide a detailed assessment on the percentage of Rh2 elimination by different routes. Some elimination routes could be supported by the reported facts or data. After I.V. administration, the simulated biliary excretion is 28%, the same as the reported value (Gu et al., 2009). The excretion rate constant of Rh2 into faeces is zero, which is in accordance with the reported faeces excretion of Rh2 in rats (Gu et al., 2009). The other unknown metabolism route in plasma contributed to about 70% elimination of Rh2, which may be correlated with oxygenation (Qian et al., 2005). In addition, the predicted concentration time profiles of Rh2 and PPD could describe the observed data at 20 mg/kg (these data were not used in model building). These results supported the rationality of the hypothesis of deglycosylation kinetics.

The study predicted the percentage of Rh2 entering the systemic circulation in PPD form. In total, 11 and 20% of the administered Rh2 were predicted to be transformed into PPD and enter the systemic circulation after I.V. administration and P.O. administration respectively. The predicted percentage was relatively reliable because it was determined by the exposure of PPD in plasma. The second peak of PPD was high and considered as the main transformation from Rh2 (route B). The first peak of PPD of I.V. administration was assumed to be caused by the transformation from Rh2 to PPD in the liver (route A). The first peak of PPD of P.O. administration might be caused by the transformation from Rh2 to PPD in the stomach (route C) (Bae et al., 2004), but route A and C only contributed to the formation of PPD slightly since the first peak was low.

Most of the administered Rh2 were predicted to be eliminated by other unknown routes. The elimination of Rh2 by other unknown routs was evaluated in the stomach by matching the pharmacokinetic profile of PPD after oral administration of Rh2. We have considered three possible mechanisms to explain the elimination of Rh2 in the stomach by the following unknown routes: 1) Rh2 is absorbed into the liver and further metabolised there; 2) Rh2 is eliminated directly in the stomach; 3) Rh2 is transformed into PPD, and later eliminated in the stomach and intestine. Although all the assumptions could render similar performance and describe the observed data well, the third mechanism seems to be more biologically feasible. It should be noted that the details of other unknown routes should be used carefully since the assumption of an unknown route was not fully validated. But the predicted percentage of other unknown routes might be valuable for the next study of mass balance.

The developed models have potential to be applied for the pharmacokinetic study of other glycosides. Glycosides are popularly applied in food and medicine as important naturally occurring substances and include hormones, sweeteners, alkaloids, flavonoids, antibiotics, etc. (Khan et al., 2017; Momtazi-Borojeni et al., 2017; Osman et al., 2017; Botelho et al., 2019; Bundgaard Anker et al., 2019; Liu et al., 2019). Most glycosides have similar pharmacokinetics characteristics with Rh2. They can be hydrolysed into active aglycons in a biological body by the glycosidase in intestine microflorae (Tribolo et al., 2007; Winotapun et al., 2013; Mishra and Aeri, 2017). Herein, it is necessary to simultaneously investigate the kinetics of the glycoside and its aglycone for the pharmacokinetic study of glycoside. When the concentrations versus time data of glycoside and its aglycone are available, the developed models in this study may be a good starting point for pharmacokinetic modelling and simulation, which is the basis for further PK/PD modelling.

**FIGURE 4:**
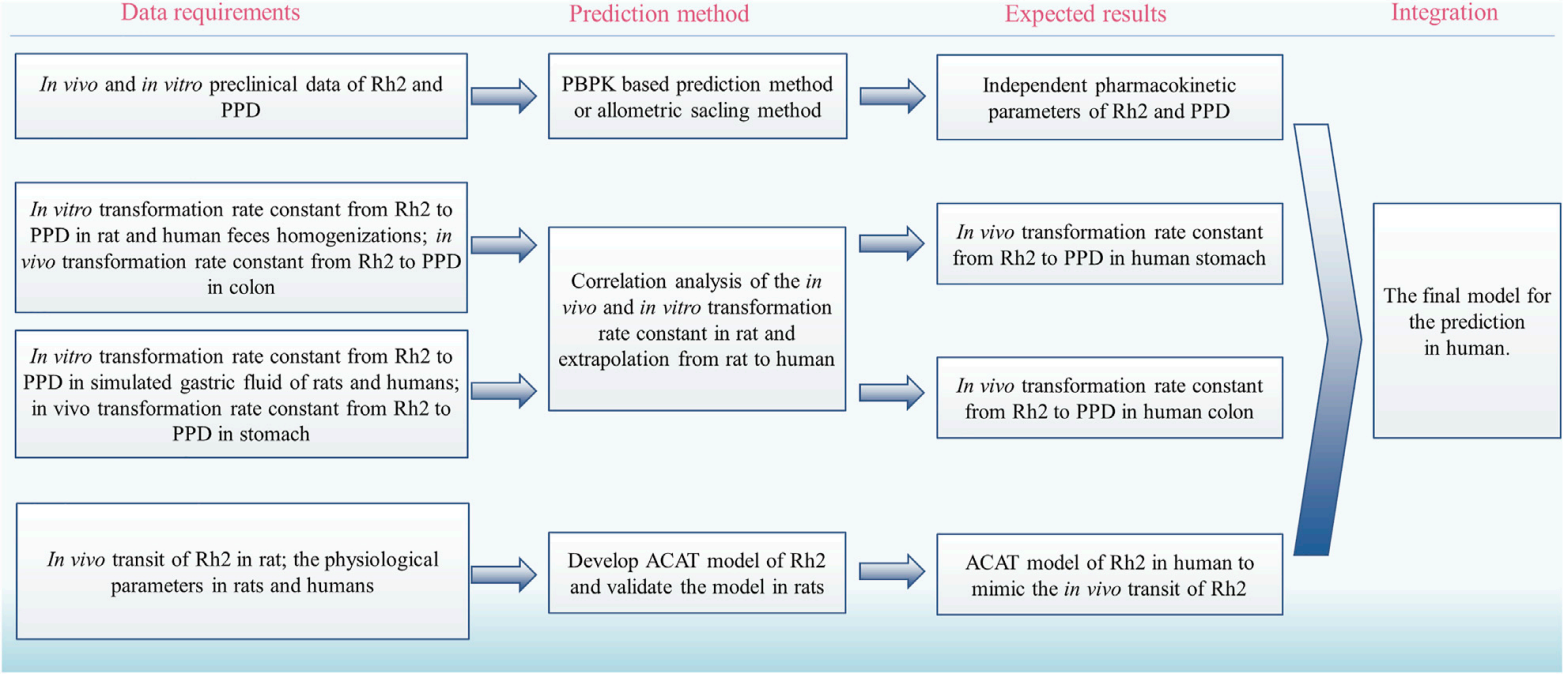
The proposed strategy to translate the deglycosylation kinetics from rats to humans. PBPK, physiologically based pharmacokinetic; ACAT, advanced compartmental absorption and transit.

The deglycosylation metabolism of Rh2 could be observed in the rat and human intestinal bacteria indicating that the developed models had the possibility to translate preclinical findings into clinical practice (Bae et al., 2004; Qian and Cai, 2010). Considering the requirements on the quantitative translation, we proposed a strategy to translate the deglycosylation kinetics from rats to humans (Figure 4):

(i) Use the reported method to predict the human pharmacokinetics of the parent drug after dosing of Rh2 and PPD, respectively, for the pharmacokinetic parameters of Rh2 and PPD, such as V_Rh2, plasma_ and V_PPD, plasma_ (Ren et al., 2019).
(ii) Find the *in vitro* and *in vivo* correlation on the transformation rate constant from Rh2 to PPD in the colon and stomach in rats.
(iii) Use the above relationship of *in vivo* and *in vitro* data to predict the human *in vivo* transformation rate constant from Rh2 to PPD based on the *in vitro* transformation rate constant from Rh2 to PPD in human faeces homogenates and simulated gastric fluid of humans, respectively.
(iv) Develop the advanced compartmental absorption and transit (ACAT) model of Rh2 in rats and extrapolate the model from rats to humans (Gobeau et al., 2016).
(v) Integrate the human pharmacokinetic parameters of Rh2 and PPD, transformation rate constants from Rh2 to PPD, and the intestinal transit of Rh2 to quantitatively predict the PK profiles of Rh2 and PPD in humans.

However, there are three key issues, which need more research: 1) the Rh2 may have better oral absorption in human than rats due to the longer intestine length and larger lumen area; 2) the extrapolation of excretion by faeces from rats to humans is challenging; 3) the proposed method of prediction needs to be validated by the actual data; but there are few publications involving the Rh2 and PPD kinetics in humans. There are some challenges regarding the interspecies differences in deglycosylation activity of the colonic microbiota and large inter-individual differences in humans (Brody, 2020). However, the proposed strategy made the first step of this long journey to translate the deglycosylation kinetics from rats to humans and more studies are warranted.

## 5 Conclusion

This study has identified that PPD plays a critical role in the pharmacokinetics of Rh2. A mechanism-based pharmacokinetic model of Rh2 was developed to quantitatively describe the kinetics of Rh2 and PPD. The percentage of transformation from Rh2 to PPD could be predicted based on the model. The developed model has the potential to be used to describe the deglycosylation kinetics of other glycosides, the PK/PD analysis of ginseng, and the herb-drug interaction between ginseng and other P-gp substrates.

## Data Availability

The original contributions presented in the study are included in the article/Supplementary Material, further inquiries can be directed to the corresponding authors.

## References

[B1] Akao T. Kanaoka M. Kobashi K. (1998). Appearance of Compound K, a Major Metabolite of Ginsenoside Rb1 by Intestinal Bacteria, in Rat Plasma after Oral Administration-Mmeasurement of Compound K by Enzyme Immunoassay. Biol. Pharm. Bull. 21 (3), 245–249. 10.1248/bpb.21.245 9556154

[B2] Alolga R. N. Nuer-Allornuvor G. F. Kuugbee E. D. Yin X. Ma G. (2020). Ginsenoside Rg1 and the Control of Inflammation Implications for the Therapy of Type 2 Diabetes: A Review of Scientific Findings and Call for Further Research. Pharmacol. Res. 152, 104630. 10.1016/j.phrs.2020.104630 31911245

[B3] Bae E. A. Han M. J. Kim E. J. Kim D. H. (2004). Transformation of Ginseng Saponins to Ginsenoside Rh2 by Acids and Human Intestinal Bacteria and Biological Activities of Their Transformants. Arch. Pharm. Res. 27 (1), 61–67. 10.1007/bf02980048 14969341

[B4] Botelho A. F. M. Pierezan F. Soto-Blanco B. Melo M. M. (2019). A Review of Cardiac Glycosides: Structure, Toxicokinetics, Clinical Signs, Diagnosis and Antineoplastic Potential. Toxicon 158, 63–68. 10.1016/j.toxicon.2018.11.429 30529380

[B5] Bridgeman S. C. Northrop W. Melton P. E. Ellison G. C. Newsholme P. Mamotte C. D. S. (2020). Butyrate Generated by Gut Microbiota and its Therapeutic Role in Metabolic Syndrome. Pharmacol. Res. 160, 105174. 10.1016/j.phrs.2020.105174 32860943

[B6] Brody H. (2020). The Gut Microbiome. Nature 577 (7792), S5. 10.1038/d41586-020-00194-2 31996824

[B7] Bundgaard Anker C. C. Rafiq S. Jeppesen P. B. (2019). Effect of Steviol Glycosides on Human Health with Emphasis on Type 2 Diabetic Biomarkers: A Systematic Review and Meta-Analysis of Randomized Controlled Trials. Nutrients 11 (9), 1965. 10.3390/nu11091965 PMC6770957 31438580

[B8] Choi M. S. Yu J. S. Yoo H. H. Kim D. H. (2018). The Role of Gut Microbiota in the Pharmacokinetics of Antihypertensive Drugs. Pharmacol. Res. 130, 164–171. 10.1016/j.phrs.2018.01.019 29391236

[B9] Dabek M. McCrae S. I. Stevens V. J. Duncan S. H. Louis P. (2008). Distribution of Beta-Glucosidase and Beta-Glucuronidase Activity and of Beta-Glucuronidase Gene Gus in Human Colonic Bacteria. FEMS Microbiol. Ecol. 66 (3), 487–495. 10.1111/j.1574-6941.2008.00520.x 18537837

[B10] Dey P. (2019). Gut Microbiota in Phytopharmacology: A Comprehensive Overview of Concepts, Reciprocal Interactions, Biotransformations and Mode of Actions. Pharmacol. Res. 147, 104367. 10.1016/j.phrs.2019.104367 31344423

[B11] Doonan S. Ho T. K. Pearce F. L. Slaughter C. A. (1978). Glycosidases in the Submaxillary Gland of the Mouse: Sexual Dimorphism and the Effects of Testosterone. Int. J. Biochem. 9 (4), 235–238. 10.1016/0020-711x(78)90004-6 648705

[B12] Feng W. Ao H. Peng C. Yan D. (2019). Gut Microbiota, a New Frontier to Understand Traditional Chinese Medicines. Pharmacol. Res. 142, 176–191. 10.1016/j.phrs.2019.02.024 30818043

[B13] Future Market Insights (2020). Global Ginseng Market: Forecast, Trend, Analysis & Competition Track - Global Review 2019 to 2027. Available at: https://www.futuremarketinsights.com/reports/ginseng-market (accessed Nov 24, 2020).

[B14] Gloster T. M. Turkenburg J. P. Potts J. R. Henrissat B. Davies G. J. (2008). Divergence of Catalytic Mechanism within a Glycosidase Family Provides Insight into Evolution of Carbohydrate Metabolism by Human Gut flora. Chem. Biol. 15 (10), 1058–1067. 10.1016/j.chembiol.2008.09.005 18848471 PMC2670981

[B15] Gobeau N. Stringer R. De Buck S. Tuntland T. Faller B. (2016). Evaluation of the GastroPlus™ Advanced Compartmental and Transit (ACAT) Model in Early Discovery. Pharm. Res. 33 (9), 2126–2139. 10.1007/s11095-016-1951-z 27278908

[B16] Gong X. Li X. Bo A. Shi R. Y. Li Q. Y. Lei L. J. (2020). The Interactions between Gut Microbiota and Bioactive Ingredients of Traditional Chinese Medicines: A Review. Pharmacol. Res. 157, 104824. 10.1016/j.phrs.2020.104824 32344049

[B17] Gu Y. Wang G. J. Sun J. G. Jia Y. W. Wang W. Xu M. J. (2009). Pharmacokinetic Characterization of Ginsenoside Rh2, an Anticancer Nutrient from Ginseng, in Rats and Dogs. Food Chem. Toxicol. 47 (9), 2257–2268. 10.1016/j.fct.2009.06.013 19524010

[B18] Gu Y. Wang G. J. Wu X. L. Zheng Y. T. Zhang J. W. Ai H. (2010). Intestinal Absorption Mechanisms of Ginsenoside Rh2: Stereoselectivity and Involvement of ABC Transporters. Xenobiotica 40 (9), 602–612. 10.3109/00498254.2010.500744 20608841

[B19] Jeong D. Ham J. Park S. Kim H. W. Kim H. Ji H. W. (2019). Ginsenoside Rh2 Suppresses Breast Cancer Cell Proliferation by Epigenetically Regulating the Long Noncoding RNA C3orf67-AS1. Am. J. Chin. Med. 47 (7), 1643–1658. 10.1142/S0192415X19500848 31645124

[B20] Jia Q. Wang L. Zhang X. Ding Y. Li H. Yang Y. (2020). Prevention and Treatment of Chronic Heart Failure through Traditional Chinese Medicine: Role of the Gut Microbiota. Pharmacol. Res. 151, 104552. 10.1016/j.phrs.2019.104552 31747557

[B21] Khan H. Khan Z. Amin S. Mabkhot Y. N. Mubarak M. S. Hadda T. B. (2017). Plant Bioactive Molecules Bearing Glycosides as Lead Compounds for the Treatment of Fungal Infection: A Review. Biomed. Pharmacother. 93, 498–509. 10.1016/j.biopha.2017.06.077 28675856

[B22] Laparra J. M. Sanz Y. (2010). Interactions of Gut Microbiota with Functional Food Components and Nutraceuticals. Pharmacol. Res. 61 (3), 219–225. 10.1016/j.phrs.2009.11.001 19914380

[B23] Lee W. K. Kao S. T. Liu I. M. Cheng J. T. (2007). Ginsenoside Rh2 Is One of the Active Principles of Panax Ginseng Root to Improve Insulin Sensitivity in Fructose-Rich Chow-Fed Rats. Horm. Metab. Res. 39 (5), 347–354. 10.1055/s-2007-976537 17533576

[B24] Liu X. Gao C. Liu X. Gao T. (2019). Efficacy and Safety of Tripterygium Glycosides for Graves Ophthalmopathy: A Systematic Review and Meta-Analysis. Medicine (Baltimore) 98 (50), e18242. 10.1097/MD.0000000000018242 31852090 PMC6922466

[B25] Lu C. Wang Y. Lv J. Jiang N. Fan B. Qu L. (2018). Ginsenoside Rh2 Reverses Sleep Deprivation-Induced Cognitive Deficit in Mice. Behav. Brain Res. 349, 109–115. 10.1016/j.bbr.2018.03.005 29544964

[B26] Mishra S. Aeri V. (2017). Biotransformation of Lignan Glycoside to its Aglycone by Woodfordia Fruticosa Flowers: Quantification of Compounds Using a Validated HPTLC Method. Pharm. Biol. 55 (1), 360–366. 10.1080/13880209.2016.1238948 27931157 PMC6130600

[B27] Momtazi-Borojeni A. A. Esmaeili S. A. Abdollahi E. Sahebkar A. (2017). A Review on the Pharmacology and Toxicology of Steviol Glycosides Extracted from Stevia rebaudiana. Curr. Pharm. Des. 23 (11), 1616–1622. 10.2174/1381612822666161021142835 27784241

[B28] Osman M. H. Farrag E. Selim M. Osman M. S. Hasanine A. Selim A. (2017). Cardiac Glycosides Use and the Risk and Mortality of Cancer; Systematic Review and Meta-Analysis of Observational Studies. PLoS One 12 (6), e0178611. 10.1371/journal.pone.0178611 28591151 PMC5462396

[B29] Peng B. He R. Xu Q. Yang Y. Hu Q. Hou H. (2019). Ginsenoside 20(S)-Protopanaxadiol Inhibits Triple-Negative Breast Cancer Metastasis *In Vivo* by Targeting EGFR-Mediated MAPK Pathway. Pharmacol. Res. 142, 1–13. 10.1016/j.phrs.2019.02.003 30735802

[B30] Popovich D. G. Kitts D. D. (2002). Structure-Function Relationship Exists for Ginsenosides in Reducing Cell Proliferation and Inducing Apoptosis in the Human Leukemia (THP-1) Cell Line. Arch. Biochem. Biophys. 406 (1), 1–8. 10.1016/s0003-9861(02)00398-3 12234484

[B31] Qi Z. Li W. Tan J. Wang C. Lin H. Zhou B. (2019). Effect of Ginsenoside Rh2 on Renal Apoptosis in Cisplatin-Induced Nephrotoxicity *In Vivo* . Phytomedicine 61, 152862. 10.1016/j.phymed.2019.152862 31048124

[B32] Qian T. Cai Z. (2010). Biotransformation of Ginsenosides Rb1, Rg3 and Rh2 in Rat Gastrointestinal Tracts. Chin. Med. 5, 19. 10.1186/1749-8546-5-19 20504301 PMC2887866

[B33] Qian T. Cai Z. Wong R. N. Jiang Z. H. (2005). Liquid Chromatography/Mass Spectrometric Analysis of Rat Samples for *In Vivo* Metabolism and Pharmacokinetic Studies of Ginsenoside Rh2. Rapid Commun. Mass. Spectrom. 19 (23), 3549–3554. 10.1002/rcm.2232 16261639

[B34] Ren H. C. Sai Y. Chen T. (2019). Evaluation of Generic Methods to Predict Human Pharmacokinetics Using Physiologically Based Pharmacokinetic Model for Early Drug Discovery of Tyrosine Kinase Inhibitors. Eur. J. Drug Metab. Pharmacokinet. 44 (1), 121–132. 10.1007/s13318-018-0496-4 30039459

[B35] Ren H. C. Sun J. G. Wang G. J. A J-Y. Xie H-T. Zha W. B. (2008). Sensitive Determination of 20(S)-Protopanaxadiol in Rat Plasma Using HPLC-APCI-MS: Application of Pharmacokinetic Study in Rats. J. Pharm. Biomed. Anal. 48 (5), 1476–1480. 10.1016/j.jpba.2008.09.045 19022601

[B36] Savage N. (2020). The Complex Relationship between Drugs and the Microbiome. Nature 577 (7792), S10–S11. 10.1038/d41586-020-00196-0 31996826

[B37] Tribolo S. Berrin J. G. Kroon P. A. Czjzek M. Juge N. (2007). The crystal Structure of Human Cytosolic Beta-Glucosidase Unravels the Substrate Aglycone Specificity of a Family 1 Glycoside Hydrolase. J. Mol. Biol. 370 (5), 964–975. 10.1016/j.jmb.2007.05.034 17555766

[B38] Wang D. D. Zhang S. (2012). Standardized Visual Predictive Check Versus Visual Predictive Check for Model Evaluation. J. Clin. Pharmacol. 52 (1), 39–54. 10.1177/0091270010390040 21257797

[B39] Wang M. Yan S. J. Zhang H. T. Li N. Liu T. Zhang Y. L. (2017). Ginsenoside Rh2 Enhances the Antitumor Immunological Response of a Melanoma Mice Model. Oncol. Lett. 13 (2), 681–685. 10.3892/ol.2016.5490 28356946 PMC5351349

[B40] Wang Y. Dong J. Liu P. Lau C. W. Gao Z. Zhou D. (2014). Ginsenoside Rb3 Attenuates Oxidative Stress and Preserves Endothelial Function in Renal Arteries from Hypertensive Rats. Br. J. Pharmacol. 171 (13), 3171–3181. 10.1111/bph.12660 24571453 PMC4080972

[B41] Winotapun W. Opanasopit P. Ngawhirunpat T. Rojanarata T. (2013). One-Enzyme Catalyzed Simultaneous Plant Cell Disruption and Conversion of Released Glycoside to Aglycone Combined with *In Situ* Product Separation as green One-Pot Production of Genipin from Gardenia Fruit. Enzyme Microb. Technol. 53 (2), 92–96. 10.1016/j.enzmictec.2013.05.001 23769308

[B42] Yue S. J. Wang W. X. Yu J. G. Chen Y. Y. Shi X. Q. Yan D. (2019). Gut Microbiota Modulation with Traditional Chinese Medicine: A System Biology-Driven Approach. Pharmacol. Res. 148, 104453. 10.1016/j.phrs.2019.104453 31541688

[B43] Zhang J. Li W. Yuan Q. Zhou J. Zhang J. Cao Y. (2019). Transcriptome Analyses of the Anti-Proliferative Effects of 20(S)-Ginsenoside Rh2 on HepG2 Cells. Front. Pharmacol. 10, 1331. 10.3389/fphar.2019.01331 31780945 PMC6855211

[B44] Zhang J. Liu M. Huang M. Chen M. Zhang D. Luo L. (2019). Ginsenoside F1 Promotes Angiogenesis by Activating the IGF-1/IGF1R Pathway. Pharmacol. Res. 144, 292–305. 10.1016/j.phrs.2019.04.021 31048033

[B45] Zhang J. Zhou F. Wu X. Gu Y. Ai H. Zheng Y. (2010). 20(S)-Ginsenoside Rh2 Noncompetitively Inhibits P-Glycoprotein *In Vitro* and *In Vivo*: A Case for Herb-Drug Interactions. Drug Metab. Dispos 38 (12), 2179–2187. 10.1124/dmd.110.034793 20837659

[B46] Zheng M. M. Xu F. X. Li Y. J. Xi X. Z. Cui X. W. Han C. C. (2017). Study on Transformation of Ginsenosides in Different Methods. Biomed. Res. Int. 2017, 8601027. 10.1155/2017/8601027 29387726 PMC5745656

[B47] Zhu Y. Zhu C. Yang H. Deng J. Fan D. (2020). Protective Effect of Ginsenoside Rg5 against Kidney Injury via Inhibition of NLRP3 Inflammasome Activation and the MAPK Signaling Pathway in High-Fat Diet/Streptozotocin-Induced Diabetic Mice. Pharmacol. Res. 155, 104746. 10.1016/j.phrs.2020.104746 32156650

[B48] Zimmermann M. Zimmermann-Kogadeeva M. Wegmann R. Goodman A. L. (2019). Mapping Human Microbiome Drug Metabolism by Gut Bacteria and Their Genes. Nature 570 (7762), 462–467. 10.1038/s41586-019-1291-3 31158845 PMC6597290

