# Supplementary material for: Mechanism-Based Pharmacokinetic Model for the Deglycosylation Kinetics of 20(S)-Ginsenosides Rh2
Source: Front Pharmacol. 2022 May 25;13:804377. doi: 10.3389/fphar.2022.804377 (PMC9175024; doi:10.3389/fphar.2022.804377)
Supplement: Supplementary file 1 [file DataSheet2.pdf]

frontiers

Supplementary Material

1. Method validation of sample analysis

The revised method was summarized as follows: A HMPL–MS was used for the concentration determination with A Shimadzu 2010A liquid chromatograph–mass spectrometer (Shimadzu, Kyoto, Japan) equipped with an APCI ionization interface used to generate positive ions [M-H2O+H]+ as reported. The compounds were separated on a reversed-phase Gemini-C18 column (150mm×4.6mm i.d., 5μm, Phenomenex, Torrance, CA, USA) with an isocratic mobile phase consisting of methanol and purified water (90:10%, v/v). The mobile phase was eluted at 0.75 mL/min using two Shimadzu LC-10ADvp pumps. The column was maintained at 40 ^{o}C. The probe voltage was operated at 4.0 kV and the temperature at 380 ℃ in APCI source. The other MS parameters were selected as followed: CDL (curved desolvation line) temperature, 250 ^{o}C; the block temperature, 200 ℃; detector gain, 1.6 kV; CDL voltage, 5 V; Q-array DC (direct current) voltage, 0 V. Nitrogen served as nebulizer gas (flow rate: 2.5 L/min) and curtain gas (pressure: 10 kPa). Mass spectra were obtained at a dwell time of 0.2 and 1s for SIM and scan mode, respectively. Quantification was performed by selected ion monitoring (SIM) of the same product ion at m/z 425.4 for Rh2 and PPD, using the internal standard method with peak area ratios.

The validation parameters of sample analysis included selectivity, precision, and accuracy. Ten batches of blank heparinized rat plasma were screened to determine the specificity. The intra- and inter-precision and accuracy of the assay validation were estimated using the inverse prediction of the concentration of the quality controls from the calibration curve.

The retention times of Rh2, PPD, and PD were approximately 7.2, 10.8, and 13min respectively. The calibration curve of Rh2 gave a reliable response from 5 to 1000 ng/mL. The mean equation of the regression line was y = 0.00485x+0.0044 (slope range, 0.00461–0.00500; intercept range, −0.0056 to −0.0023; $r^2 > 0.996$). The calibration curve of PPD provided a reliable response from 2 to 1000 ng/mL. The mean equation of the regression line was y = 0.0033x−0.00169 (slope range, 0.00310–0.00359; intercept range, 0.00109–0.0208; $r^2 > 0.996$). The limit of quantification (LOQ) of Rh2 and PPD was found to be 2 and 5 ng/mL for PPD with the signal-to-noise ration of over 5 respectively.

The intra- and inter-day precision and accuracy of our method for Rh2 and PPD are listed in Table S1. The coefficients of variation of the intra- and inter-day precisions for Rh2 validation were less than 6.65% and 9.14%, respectively. The coefficients of variation of the intra- and inter-day precisions for PPD validation were less than 7.58% and 8.89% respectively. The accuracy for Rh2 and PPD were from 98% to 107% and 94% to 109% from respectively.

Table S1 Inter-day (n =5) and Intra-day (n = 5) precision and accuracy of the intra-day Rh2 and PPD assay (n=5)

Spiked	Intra-day			Inter-day		
(ng/mL)	Measured (ng/mL) Mean±SD	CV (%)	Accuracy (%)	Measured (ng/mL) Mean±SD	CV (%)	Accuracy (%)
Rh2						
5	5.25±0.35	6.65	105.16	4.90±0.41	8.37	98.04
10	10.61±0.67	6.57	106.20	10.41±0.97	9.14	101.76
100	99.59±3.68	3.70	99.59	102.06±4.43	4.34	102.06
1000	1034.38±28.35	2.74	103.44	1035.90±41.73	4.03	103.59

PPD						
2	2.15±0.12	5.71	107.43	2.16±0.13	5.95	108.06
5	5.02±0.38	7.58	100.53	5.20±0.46	8.89	104.02
100	47.73±1.64	3.45	94.85	48.72±1.95	4.00	97.44
1000	1008.01±69.20	6.87	100.80	1023.44±51.76	5.06	102.34

2. Model evaluation

The model was evaluated and determined based on successful convergence, objective function value, parameter precisions, visual inspection of the goodness-of-fit plots and visual predictive check (VPC). Here, we showed the visual inspection of the goodness-of-fit as plots of observed data vs population predictions and conditional weighted residuals (CWRES) vs population predictions. As showed in the top panel of Figure S1, the model predicted concentrations match the observed data by visual inspection since the majority of observations being evenly distributed around the line of identity. Inspection of the figure in the bottom panel shows that data were evenly distributed about CWRES = 0 (see the trends as given by the blue line) and concentrated between CWRES = -2 and CWRES = +2. The red line (with its negative reflection) did not show any fanning, indicating no bias in the structural model.

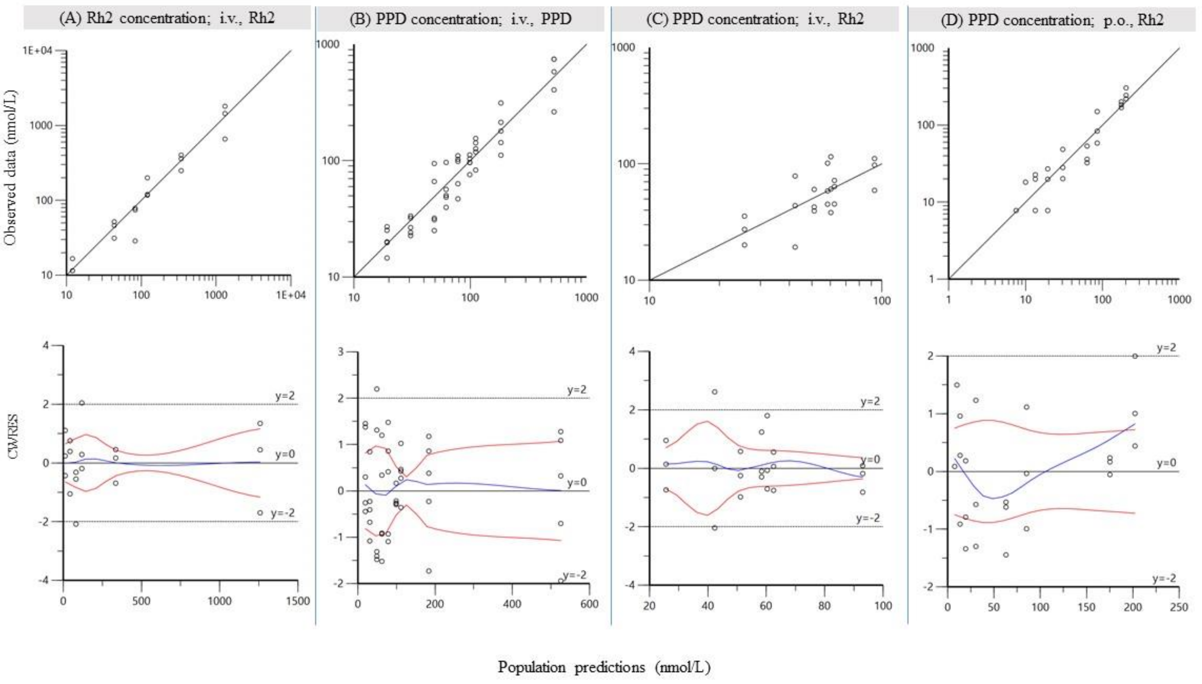

Figure S1. Goodness-of-fit plot in population pharmacokinetic model evaluation. X-axis is population predictions, and Y-axis is observed data in top panel with a line of identity (y = x). X-axis is population predictions, and Y-axis is conditional weighted residuals (CWRES) in bottom panel.

3. Weight selection

Weight selection was evaluated comparing the minimum value of the objective function provided by NLME, and approximately equal to −2×Log(likelihood) (-2LL) between two nested model, where a decrease in −2×Log(likelihood) of 3.84 is considered significant at 5% levels. If there is no significant difference, the weight should be selected with lower Akaike Information Criterion (AIC) and Bayesian Information Criterion (BIC).

The comparison of weight was showed by the indexes of LogLik, -2LL, AIC, and BID in the Table S1. Mulplicative is better than additive.

Table S1. The comparison of the goodness of fit using weight of additive and mulplicative

Weight	RetCode	LogLik	-2LL	AIC	BIC
Part A					
Mulplicative	1	-89	179	189	193
Additive	1	-121	241	251	256
Part B					
Mulplicative	1	-211	422	432	441
Additive	1	-255	510	520	529
Part C					
Mulplicative	1	-93	185	195	201
Additive	1	-94	187	197	202
Part D					
Mulplicative	1	-100	199	207	212
Additive	1	-105	210	220	226

4. Number of transit compartments

The goodness of fit was compared for model C and model D using 2, 3, and 4 transit compartments. As showed in the Table S2, the 2, 3 and 4 transit compartments have closely similar goodness of fit for the model C, and the sequence of goodness of fit is 2 compartments > 3 compartments > 4 compartments. The 3 and 4 compartments have closely similar goodness of fit for the model D, and the sequence of goodness of fit is 4 compartments > 3 compartments > 2 compartments. Finally, three compartments were used for the model after balancing the goodness of fit.

Table S2. The comparison of the goodness of fit using different number of transit compartments for model C and model D.

Transit compartment	RetCode	LogLik	-2LL	AIC	BIC
Part C					
2	1	-92	184	194	200
3	1	-93	185	195	201
4	1	-94	187	197	202

Part D					
2	1	-102	204	212	216
3	1	-100	199	207	212
4	1	-98	196	204	209

5. PK parameters

A non-compartmental analysis (NCA) was performed to determine the following pharmacokinetic metrics for Rh2 and PPD: C_{max}, T_{max}, V_{ss}, and $T_{1/2}$. The NCA was performed using Phoenix 64 Winnonlin (Pharsight, a Certara™ Company, Cary, NC, US). For Rh2 the I.V. dosing option was selected, whilst the extravascular dosing option was selected for PPD. The results were showed in the Table S3.

Table S3. The pharmacokinetic parameters from non-compartment analysis

Determination of	Parameters	10 mg/kg Mean	CV (%)	20 mg/kg Mean	CV (%)
I.V. administration of Rh2					
Rh2	C_{max} (nmol/L)	1301.3	45	3614.6	77.6
	T_{max} (h)	0.25	0	0.25	0
	V_{ss} (L/kg)	17.1	86.9	20.2	77.1
	AUC_{0-t} (h·nmol/L)	1457	42.2	3850	77.5
	$t_{1/2}$ (h)	2.23	10.6	2.36	29
PPD	C_{max} (nmol/L)	94.9	22.9	174	31.7
	T_{max} (h)	6.83	85.4	6.75	88.5
	*V_{ss} (L/kg)	174	-	144	4.4
	AUC_{0-t} (h·nmol/L)	1039	37.7	2827	40.5
	**$t_{1/2}$ (h)	7.2	-	8.3	2.5
P.O. administration of Rh2					
PPD	C_{max} (nmol/L)	255	17.1	442	49.5
	T_{max} (h)	8	0	10.7	21.7
	*V_{ss} (L/kg)	24.6	13.1	72.8	-
	AUC_{0-t} (h·nmol/L)	2377	15.3	4611	44.8
	**$t_{1/2}$ (h)	2.7	8.2	3.9	-

*The V_{ss} should be impacted by the transformation from Rh2 to PPD, which may have some gaps with the actual V_{ss} after administration of PPD.

** The $t_{1/2}$ should be impacted by the transformation from Rh2 to PPD, which may have some gaps with the real $t_{1/2}$ after administration of PPD.

"-" Mean the values could not be calculated due to the irregular PK profiles.

In this above table, $t_{1/2}$ and V_{ss} of PPD are not accurate. The accurate t1/2 and Vss of PPD should be evaluated in the PK study of PPD after IV administration of PPD.

The T_{max} of Rh2 should be "0 min" theologically. Since it is not possible to collect the 0 time point, Tmax is the first time point as for the IV dosing. If we collected the time points earlier, the T_{max} should be lower and the higher C_{max} may be observed.

Please use these data carefully.

6. The code of the model A, model B, model C, and model D

Model A:

```
test(){
    deriv(A4 = - (A4 * Ke)- (A4 * K47- A7 * K74))
    urinecpt(A0 = (A4 * Ke))
    deriv(A7 = (A4 * K47- A7 * K74))
    C = A4 / V4
    dosepoint(A4, idosevar = A4Dose, infdosevar = A4InfDose, infratevar = A4InfRate)
    error(CEps = 0.302471007581253)
    observe(CObs = C * (1 + CEps))
    stparm(V4 = tvV4 * exp(nV4))
    stparm(Ke = tvKe * exp(nKe))
```

```
    stparm(K47 = tvK47 * exp(nK47))
    stparm(K74 = tvK74 * exp(nK74))
    fixef(tvV4 = c(, 1, ))
    fixef(tvKe = c(, 4.67328070684324, ))
    fixef(tvK47 = c(, 1, ))
    fixef(tvK74 = c(, 1, ))
    ranef(diag(nKe, nK47, nK74, nV4) = c(0.011821759, 8.351944E-10, 1.0856656E-08,
0.0042379799))
}
```

Model B:

```
test(){
    deriv(A5 = - (A5 * K50)- (A5 * K58- A8 * K85))
    urinecpt(A0 = (A5 * K50))
    deriv(A8 = (A5 * K58- A8 * K85))
    C5 = A5 / V5
    dosepoint(A5, idosevar = A5Dose, infdosevar = A5InfDose, infratevar = A5InfRate)
    error(C5Eps = 0.315616110865283)
    observe(CPPDObs = C5 * (1 + C5Eps))
    stparm(V5 = tvV5 * exp(nV5))
    stparm(K50 = tvK50 * exp(nK50))
```

```
	stparm(K58 = tvK58 * exp(nK58))

	stparm(K85 = tvK85 * exp(nK85))

	fixef(tvV5 = c(, 0.28676766239056, ))

	fixef(tvK50 = c(, 4.88041672610201, ))

	fixef(tvK58 = c(, 27.2550283916885, ))

	fixef(tvK85 = c(, 3.3773877626619, ))

	ranef(diag(nV5, nK50, nK58, nK85) = c(0.017672992, 0.0037340568, 0.032710701,
2.9366489E-08))

}
```

Model C:

```
test(){

# Rh2 PK part

	deriv(A4 = - (A4 * K40) - (A4 * K45) - (A4 * K43) - (A4 * K47- A7 * K74))

	deriv(A7 = (A4 * K47- A7 * K74))

	C4 = A4 / V4

	dosepoint(A4, idosevar = A4Dose, infdosevar = A4InfDose, infratevar = A4InfRate)

	error(C4Eps = 0.30198)

	observe(CRh2Obs = C4 * (1 + C4Eps))

	stparm(V4 = tvV4 * exp(nV4))

	stparm(K45 = tvK45 * exp(nK45))
```

```
    stparm(K47 = tvK47 * exp(nK47))

    stparm(K74 = tvK74 * exp(nK74))

    fixef(tvV4(freeze)   = c(, 2.38699142225862, ))

    fixef(tvK45 = c(, 0.0909419424587497, ))

    fixef(tvK47(freeze)   = c(, 2.08448940219192, ))

    fixef(tvK74(freeze)   = c(, 0.475249675532191, ))

    K43 = 1.294319092

    K40 = 4.67263489930955 - K43 - K45

    ranef(diag(nK47, nK74, nV4) (freeze)  = c(8.351944E-10, 1.0856656E-08, 0.0042379799))

    ranef(diag(nK45) = c(8.4745882E-08))


# PPD PK part

    deriv(A5 = - (A5 * K50)- (A5 * K58- A8 * K85) + (A4 * K45) + (A6 * Kt))

    deriv(A8 = (A5 * K58 - A8 * K85))

    C5 = A5 / V5

    error(C5Eps = 0.360070541493316)

    observe(CPPDObs = C5 * (1 + C5Eps))

    stparm(V5 = tvV5 * exp(nV5))

    stparm(K50 = tvK50 * exp(nK50))

    stparm(K58 = tvK58 * exp(nK58))
```

stparm(K85 = tvK85 * exp(nK85))

fixef(tvV5(freeze)   = c(, 0.28676766239056,))

fixef(tvK50(freeze)   = c(, 4.88041672610201,))

fixef(tvK58(freeze)   = c(, 27.2550283916885,))

fixef(tvK85(freeze)   = c(, 3.3773877626619,))

ranef(diag(nV5, nK50, nK58, nK85) (freeze)   = c(0.017672992, 0.0037340568, 0.032710701, 2.9366489E-08))

transit model to link the Rh2 and PPD PK

deriv(A3 = (A4 * K43)- A3 * Kt)

deriv(A10 = (A3 * Kt) - A10 * Kt)

deriv(A11 = (A10 * Kt) - A11 * Kt)

deriv(A12 = (A11 * Kt) - A12 * Kt)

deriv(A6 = (A12 * Kt) - A6 * Kt - A6 * K60)

stparm(Kt = tvKt * exp(nKt))

stparm(K60 = tvK60 * exp(nK60))

fixef(tvKt   = c(, 0.630326647067184,))

fixef(tvK60   = c(, 1.37521915015872,))

ranef(diag(nKt, nK60) = c(0.0081599026, 0.038895088))

}

Model D:

```
test(){

# Rh2 PK part

	deriv(A1 = - (A1 * K12)-  (A1 * K13) )

	deriv(A2 =  (A1 * K12)-  (A2 * Kt) -A2 * K20)

	dosepoint(A1)

	stparm(K12 = tvK12 * exp(nK12))

	stparm(K20 = tvK20 * exp(nK20))

	stparm(K13 = tvK13 * exp(nK13))

	fixef(tvK13 = c(, 0.245621156919217, ))

	fixef(tvK12 = c(, 0.15700949234679, ))

	fixef(tvK20 = c(, 29.3050253565721, ))

	ranef(diag(nK13, nK12, nK20) = c(1.6751578E-08, 0.063255146, 0.17753114))


# PPD PK part

	deriv(A5 = - (A5 * K50)- (A5 * K58- A8 * K85) + (A2 * Kt) + (A6 * Kt))

	deriv(A8 = (A5 * K58 - A8 * K85))

	C5 = A5 / V5

	error(C5Eps = 0.310893890164477)
```

observe(CPPDObs = C5 * (1 + C5Eps))

stparm(V5 = tvV5 * exp(nV5))

stparm(K50 = tvK50 * exp(nK50))

stparm(K58 = tvK58 * exp(nK58))

stparm(K85 = tvK85 * exp(nK85))

fixef(tvV5(freeze) = c(, 0.28676766239056,))

fixef(tvK50(freeze) = c(, 4.88041672610201,))

fixef(tvK58(freeze) = c(, 27.2550283916885,))

fixef(tvK85(freeze) = c(, 3.3773877626619,))

ranef(diag(nV5, nK50, nK58, nK85) (freeze) = c(0.017672992, 0.0037340568, 0.032710701, 2.9366489E-08))

transit model to link the Rh2 and PPD PK

deriv(A3 = (A1 * K13)- A3 * Kt)

deriv(A10 = (A3 * Kt) - A10 * Kt)

deriv(A11 = (A10 * Kt) - A11 * Kt)

deriv(A12 = (A11 * Kt) - A12 * Kt)

deriv(A6 = (A12 * Kt) - A6 * Kt - A6 * K60)

stparm(Kt = tvKt * exp(nKt))

stparm(K60 = tvK60 * exp(nK60))

fixef(tvKt(freeze) = c(, 0.630326647067184,))

```
fixef(tvK60(freeze)      = c(, 1.37521915015872, ))

ranef(diag(nKt, nK60) (freeze)      = c(0.0081599026, 0.038895088))

}
```
