# Supplementary material for: Mechanism-Based Pharmacokinetic Model for the Deglycosylation Kinetics of 20(S)-Ginsenosides Rh2
Source: Front Pharmacol. 2022 May 25;13:804377. doi: 10.3389/fphar.2022.804377 (PMC9175024; doi:10.3389/fphar.2022.804377)
Supplement: Supplementary file 2 [file DataSheet1.pdf]

APPENDIX A:

A.1 The final mechanism based pharmacokinetic model

The final mechanism based pharmacokinetic model is implemented to describe the plasma concentration versus time profiles of Rh2 and PPD after I.V. or oral administration of Rh2 in rats. The Equations A1~A3 describe the distribution and elimination of Rh2 in circulation system.

$$\frac{dA_{Rh2,\ plasma}}{dt} = -A_{Rh2,\ plasma} \cdot (k_{40} + k_{45} + k_{43}) - A_{Rh2,\ plasma} \cdot k_{47} + A_{Rh2,\ PC} \cdot k_{74} \quad \text{(A1)}$$

$$\frac{dA_{Rh2,\ PC}}{dt} = A_{Rh2,\ plasma} \cdot k_{47} - A_{Rh2,\ PC} \cdot k_{74} \quad \text{(A2)}$$

$$C_{Rh2,\ plasma} = A_{Rh2,\ plasma}/V_{Rh2,\ plasma} \quad \text{(A3)}$$

Where, $A_{Rh2,\ plasma}$ is the amount of Rh2 in plasma (compartment 4); $A_{Rh2,\ PC}$ is the amount of Rh2 in peripheral compartment (compartment 7); $V_{Rh2,\ plasma}$ is the volume of Rh2 in plasma (compartment 4); $C_{Rh2,\ plasma}$ is the concentration of Rh2 in plasma (compartment 4); k_{47} is the transfer rate constant of Rh2 from compartment 4 to compartment 7; k_{74} is the transfer rate constant of Rh2 from compartment 7 to compartment 4; k_{45} is the transformation rate constant from Rh2 to PPD in compartment 4; k_{43} is the excretion rate constant of Rh2 in bile; k_{40} the elimination rate constant of Rh2 by other routes in compartment 4.

The equations of A4 ~ A6 describe the absorption, formations, distribution, and elimination of PPD in plasma.

$$\frac{dA_{PPD,\ plasma}}{dt} = -A_{PPD,\ plasma} \cdot k_{50} - A_{PPD,\ plasma} \cdot k_{58} + A_{PPD,\ PC} \cdot k_{85} + A_{Rh2,\ plasma} \cdot k_{45} + A_{PPD,\ colon} \cdot k_t + A_{PPD,\ STO} \cdot k_t \quad \text{(A4)}$$

$$\frac{dA_{PPD,\ PC}}{dt} = A_{PPD,\ plasma} \cdot k_{58} - A_{PPD,\ PC} \cdot k_{85} \quad \text{(A5)}$$

$$C_{PPD,\ plasma} = A_{PPD,\ plasma}/V_{PPD,\ plasma} \quad \text{(A6)}$$

Where, $A_{PPD,\ plasma}$ is the amount of PPD in plasma (compartment 5); $A_{PPD,\ PC}$ is the amount of PPD in peripheral compartment (compartment 8); $A_{PPD,\ colon}$ is the amount of PPD in colon (compartment 6); $A_{PPD,\ STO}$ is the amount of PPD in stomach (compartment 2); $V_{PPD,\ plasma}$ is the volume of PPD in plasma (compartment 5); $C_{PPD,\ plasma}$ the concentration of PPD in plasma (compartment 5); k_{58} is the transfer rate constant of PPD from compartment 5 to compartment 8; k_{85} is the transfer rate constant of PPD from compartment 8 to compartment 5; k_{50} is the elimination rate constant of PPD in compartment 5.

The equations of A7 ~ A13 describe the transit of Rh2 in intestine system and its transformation from Rh2 to PPD in stomach and colon.

$$\frac{dA_{Rh2,\ intestine}}{dt} = A_{Rh2,\ plasma} \cdot k_{43} + A_{Rh2,\ STO} \cdot k_{13} - A_{Rh2,\ intestine} \cdot k_t \quad \text{(A7)}$$

$$\frac{dA_{T1}}{dt} = A_{Rh2,\ intestine} \cdot k_t - A_{T1} \cdot k_t \quad \text{(A8)}$$

$$\frac{dA_{T2}}{dt} = A_{T1} \cdot k_t - A_{T2} \cdot k_t \quad \text{(A9)}$$

$$\frac{dA_{T3}}{dt} = A_{T2} \cdot k_t - A_{T3} \cdot k_t \quad \text{(A10)}$$

$$\frac{dA_{PPD,\ colon}}{dt} = A_{T3} \cdot k_t - A_{PPD,\ colon} \cdot k_t - A_{PPD,\ colon} \cdot k_{60} \quad \text{(A11)}$$

$$\frac{dA_{Rh2,\ STO}}{dt} = -A_{Rh2,\ STO} \cdot k_{12} - A_{Rh2,\ STO} \cdot k_{13} \quad \text{(A12)}$$

$$\frac{dA_{PPD,\ STO}}{dt} = A_{Rh2,\ STO} \cdot k_{12} - A_{PPD,\ STO} \cdot k_t - A_{PPD,\ STO} \cdot k_{20} \quad \text{(A13)}$$

Where, $A_{Rh2,\ intestine}$, $A_{Rh2,\ STO}$, and A_{T1} ~ A_{T3} are amount of Rh2 in intestine (compartment 3), stomach (compartment 4), and compartment T1~T3; $A_{PPD,\ colon}$ and $A_{PPD,\ STO}$ are the amount of PPD in colon (compartment 6) and stomach (compartment 2) respectively; k_t is the transit rate constant of Rh2 in intestine and absorption rate constant of PPD; k_{60} is the elimination rate constant of PPD in compartment 6; k_{12} is the transformation rate constant from Rh2 to PPD in stomach; k_{13} is the transit rate constant of Rh2 from stomach to intestine; k_{20} is the elimination rate constant of PPD in stomach.

A.2 Model reduction for parameter estimation

Based on the information of drug administration, the full mechanistic model can be reduced into four sub-model for the purpose of model parameter estimation:

A) A two-compartment I.V. pharmacokinetic model (Figure A1, sub-model A) describing the time course of Rh2 after I.V. administration of Rh2, with two Rh2-related states (i.e. $A_{Rh2,\ plasma}$ and $A_{Rh2,\ PC}$);

B) A two-compartment I.V. pharmacokinetic model (Figure A1, sub-model B) describing the time course of PPD after I.V. administration of PPD, with two PPD-related states (i.e. $A_{PPD,\ plasma}$ and $A_{Rh2,\ PC}$);

C) A model for the kinetic of PPD after I.V. administration of Rh2 (Figure A1, sub-model C). This model includes the *in vivo* disposition and transformation of Rh2 and PPD (sub-model A and B), the transit of Rh2 in intestine ($A_{Rh2,\ intestine}$ and $A_{Rh2,\ T1\sim T3}$)and the transformation of PPD in colon ($A_{PPD,\ colon}$).

D) A model for the kinetic of PPD after oral administration of Rh2 (Figure A1, sub-model D). This model includes the transformation from Rh2 to PPD in stomach, transit of Rh2 in intestine, the transformation of PPD in colon and the absorption of PPD in intestine and colon. The sub-model D shares the same framework with sub-model C concerning with *in vivo* disposition of PPD (sub model B), the transit of Rh2 in intestine, and the transformation of PPD in colon.

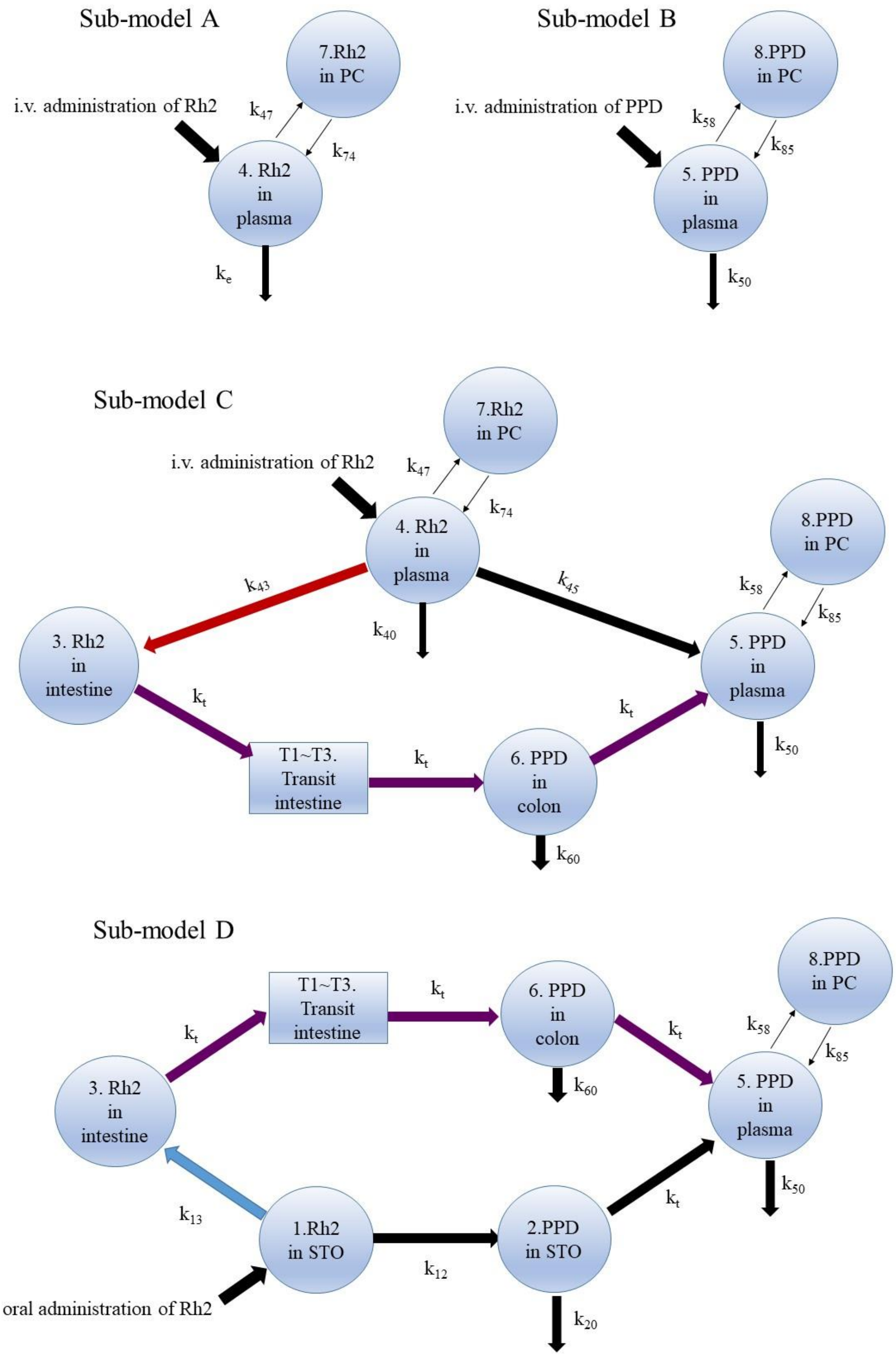

Figure A1. The framework of the sub-model A, B, C and D.

A.2.1 Characterization of Rh2 pharmacokinetics (sub-model A)

The divided sub-model A is classic I.V. two-compartment model, which is used to characterize the concentration-time profiles for Rh2 following I.V. bolus administration of Rh2.

The steps for the estimation of model parameters of Part A are described as follows:

Let

$$k_e = k_{43} + k_{45} + k_{40} \qquad \text{(A14)}$$

Where, K_e is the total elimination rate constant of Rh2 in compartment 4.

Then the A1 could be simplified as the A15:

$$\frac{dA_{Rh2,\ plasma}}{dt} = -A_{Rh2,\ plasma} \cdot k_e - A_{Rh2,\ plasma} \cdot k_{47} + A_{Rh2,\ PC} \cdot k_{74} \qquad \text{(A15)}$$

The concentrations versus time data of Rh2 (I.V., 10 mg/kg) were used to get the optimized fixed effect and random effect of k_{47}, k_{74}, k_e, and $V_{Rh2,\ plasma}$ by fitting the sub-model A with equations of A15, A2, and A3.

A.2.2 Characterization of PPD pharmacokinetics (sub-model B)

The divided sub-model B is classic I.V. two-compartment model, which is used to characterize the concentration-time profiles for PPD following I.V. bolus administration of PPD. The reported concentrations versus time data of PPD (I.V., 0.2 mg/kg) (Ren et al.,

2008) were used to fit the sub-model B with equations of A4 ~ A6 and get the optimized parameter values including $V_{PPD,\ plasma}$, k_{50}, k_{58}, and k_{85}.

A.2.3 Characterization of PPD kinetics after I.V. administration of Rh2 (sub-model C)

After I.V. Rh2 administration, Rh2 was transformed to PPD in circulation system with transformation rate constant k_{45} and excreted into bile with excretion rate constant of k_{43}. The excreted Rh2 transited from intestine to colon through three transit compartments and metabolized to PPD in colon. The transformed PPD could be re-absorbed into circulation system. After I.V. administration of Rh2, the Rh2 should not be observed in stomach (compartment 1), as well as PPD (compartment 2). Herein, the A4 and A7 can be simplified as A16 and A17 respectively:

$$\frac{dA_{PPD,\ plasma}}{dt} = -A_{PPD,\ plasma} \cdot k_{50} - A_{PPD,\ plasma} \cdot k_{58} + A_{PPD,\ PC} \cdot k_{85} + A_{Rh2,\ plasma} \cdot k_{45} + A_{PPD,\ colon} \cdot k_t \quad \text{(A16)}$$

$$\frac{dA_{Rh2,\ intestine}}{dt} = A_{Rh2,\ plasma} \cdot k_{43} - A_{Rh2,\ intestine} \cdot k_t \quad \text{(A17)}$$

The k_{43} was estimated by the following equation:

$$k_{43} = k_e \cdot \text{biliary excretion ratio of Rh2} \quad \text{(A18)}$$

Where, the biliary excretion ratio of Rh2 is the 28% (Gu et al., 2009).

The Rh2 and PPD related parameters (sub-model A and B) were frozen to further optimize the sub-model C related parameters, including k_{40}, k_{45}, and k_t, as well as the number of transit compartments by fitting the sub-model C with equations of A1 ~ A3, A5, A6, A8 ~

A11, and A16 ~ A18 using the concentrations versus time data of PPD after I.V. administration of Rh2 at 10 mg/kg.

A.2.4 Characterization of PPD kinetics after oral administration of Rh2 (sub-model D)

After oral administration of Rh2, little Rh2 should be observed in plasma (compartment 4) and peripheral compartment (compartment 7) due to the poor bioavailability. Herein, the A4 and A7 can be simplified as A19 and A20 respectively:

$$\frac{dA_{PPD,\ plasma}}{dt} = -A_{PPD,\ plasma} \cdot k_{50} - A_{PPD,\ plasma} \cdot k_{58} + A_{PPD,\ PC} \cdot k_{85} + A_{PPD,\ STO} \cdot k_t + A_{PPD,\ colon} \cdot k_t \quad \text{(A19)}$$

$$\frac{dA_{Rh2,\ intestine}}{dt} = A_{Rh2,\ STO} \cdot k_{13} - A_{Rh2,\ intestine} \cdot k_t \quad \text{(A20)}$$

The PPD related parameters (sub-model C) were frozen to optimize the other sub-model D related parameters (k_{12}, k_{20}, and k_{13}) by fitting the sub-model D with equations of A1 ~ A3, A5, A6, A8 ~ A11, A19, and A20 using the concentrations versus time data of PPD after oral administration of Rh2 at 10 mg/kg.
